# Thymoquinone Inhibits Virulence Related Traits of *Cronobacter sakazakii* ATCC 29544 and Has Anti-biofilm Formation Potential

**DOI:** 10.3389/fmicb.2017.02220

**Published:** 2017-11-28

**Authors:** Chao Shi, Chunhong Yan, Yue Sui, Yi Sun, Du Guo, Yifei Chen, Tong Jin, Xiaoli Peng, Linlin Ma, Xiaodong Xia

**Affiliations:** ^1^College of Food Science and Engineering, Northwest A&F University, Yangling, China; ^2^College of Animal Science and Technology, Northwest A&F University, Yangling, China; ^3^School of Life Science and Technology, Xi'an Jiaotong University, Xi'an, China; ^4^Xi'An Yurun Agricultural Products Global Sourcing Co., LTD., Xi'an, China

**Keywords:** *Cronobacter sakazakii*, thymoquinone, sub-inhibitory concentration, HT-29 cells, virulence factors

## Abstract

The aim of this study was to determine whether thymoquinone, the principal active ingredient in the volatile oil of *Nigella sativa* seeds, could suppress certain virulence traits of *Cronobacter sakazakii* ATCC 29544 which contribute to infection. Sub-inhibitory concentrations of thymoquinone significantly decreased motility, quorum sensing, and endotoxin production of *C. sakazakii* ATCC 29544 and biofilm formation of *C. sakazakii* 7-17. Thymoquinone substantially reduced the adhesion and invasion of *C. sakazakii* ATCC 29544 to HT-29 cells and decreased the number of intracellular bacterial cells within the RAW 264.7 macrophage cells. Thymoquinone also repressed the transcription of sixteen genes involved in the virulence. These findings suggest that thymoquinone could attenuated virulence-related traits of *C. sakazakii* ATCC 29544, and its effects on other *C. sakazakii* strains and *in vivo C. sakazakii* infection need further investigation.

## Introduction

*Cronobacter* spp., formerly known as *Enterobacter sakazakii*, is a Gram-negative, non-spore-forming, rod-shaped bacterium (Fang et al., 2012). *Cronobacter* spp. has been isolated from water, sediment, soil and herbs (Iversen and Forsythe, 2004). Powdered infant formula (PIF) and powdered milk have been identified as the most common sources and vehicles of *Cronobacter* spp. transmission (Fang et al., 2012). *Cronobacter* spp. is associated with bacteraemia and sepsis, cerebrospinal and peritoneal fluid accumulation, brain abscesses, cyst formation, necrotizing enterocolitis (NEC), meningitis, and intracerebral infarctions. *Cronobacter* spp. is currently considered to consist of 7 species: *Cronobacter sakazakii, C. malonaticus, C. universalis, C. dublinensis, C. muytjensii, C. condiment*, and *C. zurichensis* (Stephan et al., 2014), among which *C. sakazaii* is one of the two group 1 clinically relevant species that form the majority of the clinical isolates.

*C. sakazakii* possesses an array of virulence factors which aid in tissue adhesion, invasion, and host cell injury (Singh et al., 2015). Previous studies have reported OmpX and OmpA, which are outer membrane proteins (OMPs) of *E. sakazakii*, are potential virulence marker (Himelright et al., 2002; Kim et al., 2010). Various plasmid associated genes such as *Cronobacter* plasminogen activator (*cpa*) and genes responsible for iron acquisition (*eit*CBAD and *iuc*ABD/*iut*A) have been reported in different strains of *Cronobacter* spp. (Stoll et al., 2004; Hunter and Bean, 2013; Singh et al., 2015). Lipopolysaccarides (LPS) is an outer membrane virulence factor of *E. sakazakii*, which interacts with enterocytes through LPS mediated binding to toll like receptor-4 (TLR4) inducing necrotizing enterocolitis in animals (Hunter et al., 2008). Quorum sensing is a process that enables bacteria to communicate and modify behavior in response to changes in the cell population density. The process involves the production, release, detection and of extracellular signaling molecules called autoinducers (Ng and Bassler, 2009). Phenotypes that are controlled by a quorum sensing system include swarming and the expression of virulence factors such as lytic enzymes, toxins, siderophores, and adhesion molecules (de Kievit and Iglewski, 2000; Miller and Bassler, 2001; Whitehead et al., 2001; De Windt et al., 2003).

With ever-increasing antimicrobial resistant strains, alternative strategies, other than antibiotics, to treat or prevent infections caused by *C. sakazakii* have been explored (Shi et al., 2016a,b). Plant materials have received a great deal of interest as an alternative method to control pathogenic microorganisms. Many studies have demonstrated that components derived from plants (such as essential oils) show antimicrobial activity against a broad spectrum of microorganisms (Cowan, 1999; Turgis et al., 2009).

The black seed (*Nigella sativa*, Ranunculaceae family), also known as Black Caraway Seed and “the Blessed Seed,” is an annual herb that grows in countries bordering the Mediterranean Sea, Pakistan and India. The seed has been used as a natural remedy for more than 2,000 years to promote health and treat diseases (Gali-Muhtasib et al., 2006). And the seeds and oil of this plant are demonstrated to have a very low degree of toxicity (Ali and Blunden, 2003). Thymoquinone (TQ), the principal active ingredient in the volatile oil of *N. sativa* seeds (El-Mahmoudy et al., 2002), has been reported to have many significant pharmacological properties, including anti-oxidant, anti-inflammatory, anti-histaminic, analgesic, anti-hypertensive, and anti-mutagenic functions (Ragheb et al., 2009). Investigators have shown that TQ also has significant anti-neoplastic effect on human pancreatic adenocarcinoma, uterine sarcoma, and leukemic cell lines, while it is minimally toxic to normal cells (Worthen et al., 1998). Forouzanfar et al. (2014) have proved that TQ has antimicrobial activity against *Staphylococcus aureus, Escherichia coli, Salmonella enteritidis, Pseudomonas aeruginosa*, and fungi.

Although, the antimicrobial activity of *N. sativa* and its extract has been extensively studied, little information is available in the literature about the anti-virulence potential of thymoquinone on *C. sakazakii*. Therefore, the aim of this study was to investigate the effect of thymoquinone on virulence-related traits of *C. sakazakii* ATCC 29544. In addition, its effect on biofilm formation was also investigated with a strong biofilm producing food-derived *C. sakazakii* isolate 7-17.

## Materials and methods

### Reagents

The TQ (Tokyo Chemical Industry Co., Tokyo, Japan; CAS: 490-91-5) stock solutions were prepared in 0.1% dimethyl sulfoxide (DMSO) before use as described in a previous study (Shi et al., 2015).

### Bacterial strains and culture conditions

*C. sakazakii* strains ATCC 29544, ATCC 29004, ATCC BAA-894, ATCC 12868 (ATCC, Manassas, USA) were used in this study. Six other *C. sakazakii* strains were taken from our laboratory strain collection, which were originally isolated from various infant formula and infant rice cereal sources in China. Since *C. sakazakii* strain ATCC 29544 showed weak biofilm production in preliminary experiments, the relatively strong biofilm-producing *C. sakazakii* strain 7-17 was selected for use in the biofilm formation study (Li et al., 2016). The QS indicator strain *Chromobacterium violaceum* ATCC 12472 (ATCC, Manassas, USA) was used for QS inhibition assays (Yang et al., 2016). All *C. sakazakii* strains were prepared as described in a previous study (Shi et al., 2015).

### Determination of minimum inhibitory concentration (MIC) of TQ

MIC was determined by agar dilution method as described in a previous study (Shi et al., 2016a). Tryptone soya agar (TSA) was aseptically transferred into sterile 24-well plates. TQ was added to the warm medium (45°C) to obtain final concentrations of 0, 200, 400, 600, 1,200, 1,800, 2,400, and 3,600 μmol/L. After hardening, the TSA was spotted with 2 μl of the *C. sakazakii* suspension (~10^5^ CFU), then the samples were incubated at 37°C for 24 h. The lowest concentration of TQ at which no visible growth of test organisms was observed, was determined as MIC. Ampicillin (100 mg/L) was used as a positive control for the test.

### Determination of sub-inhibitory concentration (SIC) of TQ

The SICs of TQ were determined by broth dilution (Li et al., 2014) with some modifications. One hundred and twenty-five microlitres of the tryptone soya broth (TSB) (with 6.0 log CFU of *C. sakazakii*) were inoculated in 96-well microtiter plate. The same volume of TQ solution [TQ dissolved in TSB contain 0.1% DMSO (v/v)] was added to the cultures to obtain final concentrations of 25, 50, 100, 200, 400, 600, 1,200, and 2,400 μmol/L. TSB containing 0.1% DMSO was used as a negative control. The samples were further cultured at 37°C, and cell growth was monitored at 600 nm using a multimode plate reader (Tecan, Infinite™ M200 PRO, Männedorf, Switzerland).

### Motility assay

Swimming and swarming were evaluated in Luria-Bertani (LB) broth containing different agar concentrations as previously described (Li et al., 2014). For analysis of swimming ability, a medium containing 20 ml of LB broth and 0.3% (wt/vol) of agar was used. TQ was added to the warm medium (45°C) to obtain final concentrations of 0, 400 and 600 μmol/L. Then the plates were allowed to dry for 1 h at 25°C before use. Five microliters of each *C. sakazakii* ATCC 29544 culture (~6.0 log CFU) were inoculated at the center of this semisolid medium and the plates were incubated at 37°C for 7 h. After this, the diameter of the bacterial halo was recorded. The medium without TQ was used as a control.

For analysis of bacterial swarming ability, a medium containing 20 ml of LB broth, 0.5% (wt/vol) agar and 0.5% (wt/vol) glucose was used. Five microliters of *C. sakazakii* culture (~6.0 log CFU) were stabbed into the semisolid medium with and without TQ at 0, 400, and 600 μmol/L. The plates were incubated upside down at 37°C for 20 h. The size of the swarm area in the presence or absence of TQ was calculated using AutoCAD.

### *C. sakazakii* ATCC 29544 endotoxin assay

ToxinSensor™ Chromogenic LAL Endotoxin Assay Kit (GenScript, Piscataway, NJ, U.S.A.) was used according to the method of Amalaradjou et al. (2014). Overnight culture of *C. sakazakii* strains ATCC 29544 was centrifuged (5,000 × g, 10 min, 4°C) and re-suspended in TSB. Then, 50 μl of the cell suspension (OD_600 nm_ = 0.5) and TQ solution was added in 30 ml TSB to obtain a final concentration of 0, 400, and 600 μmol/L. The bacterial cells were grown at 37°C to its mid-log phase. Non-inoculated TSB was used as a control. Samples were treated following manufacturer instructions and analyzed by a microplate spectrophotometer (Model 680; Bio-Rad, Hercules, CA, U.S.A.).

### Quantitative QS inhibition assay

The effect of TQ on the QS inhibitory activity was indirectly measured by quantifying the violacein production with the indicator strain *C. violaceum* ATCC 12472 (Taganna et al., 2011). First, the inhibitory concentration of TQ on the growth of *C. violaceum* ATCC 12472 was studied to determine the SIC to be used in further experiments. The two highest concentrations of TQ that did not inhibit *C. violaceum* ATCC 12472 growth after 12 h of incubation were selected as SICs for this study.

A flask incubation assay was used to quantify the QS-inhibitory activity of TQ. An overnight culture of *C. violaceum* ATCC 12472 was diluted to an OD_600 nm_ of 0.5. Volumes (30 ml) of LB broth containing different concentrations of TQ were placed into separate flasks. Each flask was inoculated with 100 μl of the culture. The flasks were incubated at 30°C for 24 h with 150 rpm. The violacein extraction and quantitation were carried out as previously described by Choo et al. (2006) with minor modifications. Briefly, 5 ml of the *C. sakazakii* culture was centrifuged (5,000 × g, 5 min, 4°C) to precipitate insoluble violacein and the culture supernatant was discarded. Then, 1 ml of DMSO was added to the pellet and the solution was vortexed vigorously for 1 min to completely solubilize violacein. The solution was then centrifuged at 5,000 × g for 10 min to remove the cells. Two hundred microlitres of the violacein-containing supernatants were added to 96-well microtiter plates and the absorbance of violacein-containing supernatants was read with a microplate spectrophotometer (Model 680; Bio-Rad, Hercules, CA, U.S.A.) at a wavelength of 585 nm.

### Specific biofilm formation (SBF) inhibition assay

Biofilm formation assays were performed according to the method described by Naves et al. (2008) with minor modifications. Overnight cultures of *C. sakazakii* strain 7-17 were centrifuged (5,000 × g, 10 min, 4°C) and re-suspended in TSB. Then, 250 μl of the cell suspension (OD_600 nm_ = 1) were inoculated in sterile 96-well microtiter plates. TQ was added to each of the wells to obtain final concentrations of 0, 400, and 600 μmol/L. Non-inoculated TSB was used as a control. The plates were incubated at 25 or 12°C for 24, 48, and 72 h without agitation. At each time point, the optical densities (ODs) of cell growth were measured at 630 nm using a microplate spectrophotometer (Model 680; Bio-Rad, Hercules, CA, U.S.A.). The suspension was then removed and the wells were rinsed once with 350 μl of distilled water. After being air dried for 30 min, the wells were stained with 250 μl of 1% crystal violet (wt/vol) (Tianjin Kermel Chemical Regent Co., Ltd, Tianjin, China) for 20 min at room temperature. To remove the non-conjugated colorant, the wells were rinsed three times with 350 μl of distilled water. After being air-dried for 30 min, the adhered dye was solubilized in 250 μl of 33% (vol/vol) glacial acetic acid and incubated for 20 min at room temperature before the ODs were read at 570 nm. The SBF was calculated by attaching and stained bacteria (OD_570 nm_) normalized with cell growth (OD_630 nm_). The experiment was replicated at least three times.

### Cell culture

The human colonic cell line HT-29 was maintained in Dulbecco's modified Eagle medium (DMEM) (Gibco, Grand Island, NY, U.S.A.). Murine macrophage cell line RAW 264.7 cells were grown in RPMI 1640 (Gibco). Both media were supplemented with 10% (vol/vol) fetal bovine serum (FBS) (Hyclone, Logan, UT, U.S.A.), 1% (vol/vol) non-essential amino acids (Gibco) and 1% (vol/vol) double antibiotic solution (100 U/ml penicillin and 100 μg/ml streptomycin; Hyclone). Maintenance of the cell lines and subsequent experiments were carried out at 37°C in a humidified atmosphere containing 5% CO_2_.

### Cell viability assay

HT-29 cells were seeded at a density of 10^5^/ml into the wells of 96-well plates and incubated at 37°C with 5% (vol/vol) CO_2_ for 24 h. To determine an appropriate non-cytotoxic concentration of TQ for use in this study, cells were grown with different concentrations of TQ and its cytotoxic effect was evaluated by 3-(4,5-dimethyl-2-thiazolyl)-2,5-diphenyl-2H-tetrazolium bromide (MTT) assay (Li et al., 2014). After incubation, cultures were removed, 200 μl of 0.5% (wt/vol) MTT dissolved in phosphate-buffered saline (PBS, 0.01 mol/L, pH = 7.4) was added, and the plates were incubated for 4 h. One hundred microlitres of DMSO was then added to each well to dissolve the formazan crystals. Absorbance at 570 nm was measured with a microplate reader microplate spectrophotometer (Bio-Rad). Cell viability was expressed as a percentage of the control (untreated cells).

### Adhesion and invasion of cells

The effect of TQ on the adhesion and invasion of *C. sakazakii* ATCC 29544 was investigated by using HT-29 cells, according to a previous study (Li et al., 2014). Trypsin-treated cells were seeded in 24-well tissue culture plates and grown in supplemented DMEM (10^5^ cells per well) for 24 h. *C. sakazakii* was grown to mid-log phase, centrifuged, and resuspended in cell culture media without double antibiotics. Then, the HT-29 cells were rinsed with PBS and inoculated with 10^8^ CFU of *C. sakazakii*, equivalent to a multiplicity of infection (MOI) of 1,000. At the same time, DMEM containing different concentrations of TQ were added to the wells. Plates were incubated at 37°C in a humidified, 5% CO_2_ incubator.

For adhesion assay, the infected monolayer cells were rinsed three times in PBS after 1 h of incubation, and lysed with 0.1% Triton X-100 (Amresco, Solon, OH, U.S.A.). The number of viable adherent *C. sakazakii* ATCC 29544 was determined by the serial dilution and plating on TSA plates and incubated at 37°C for 24 h before counting. For the invasion assay, the monolayers were incubated for 1 h following infection, rinsed three times in PBS, and incubated for another 30 min in whole media containing gentamicin (100 μg/ml; Amresco) to kill the extracellular bacteria. Finally, the wells were washed with PBS three times. And the numbers of invaded *C. sakazakii* cells were determined as described in the adhesion assay. The results were expressed as a percentage relative to that of the control group.

### Intracellular survival and replication of *C. sakazakii* ATCC 29544 in RAW 264.7 cells

The murine macrophage cell line RAW 264.7 cells were maintained in RPMI 1640 medium with 10% FBS. Twenty-four hours prior to infection, activated cells were seeded in 24-well tissue plates (10^5^ cells per well) and cultured at 37°C under 5% CO_2_. *C. sakazakii* ATCC 29544 was incubated to its mid-log phase in TSB with various concentrations of TQ (0, 400, and 600 μmol/L). Then the RAW 264.7 cells were washed gently with PBS and infected with 10^7^ CFU (100 MOI) of *C. sakazakii* ATCC 29544. The plates were incubated for 45 min at 37°C with 5% CO_2_. After incubation, RAW 264.7 cells were re-suspended in RPMI 1640 containing 1% FBS with gentamicin (100 μg/ml) and incubated at 37°C with 5% CO_2_ for 30 min.

For intracellular survival assays, the cells were then washed three times with PBS and lysed with 0.1% Triton. After dilution (PBS 1:10), the samples were enumerated on TSA plates. The results were presented as the number of intracellular *C. sakazakii* ATCC 29544 cells after TQ treatment. For replication assays, each well-containing bacterial cells was replenished with RPMI 1640 containing 1% FBS with gentamicin (10 μg/ml) and incubated at 37°C with 5% CO_2_ for either 24 or 48 h. Cell washing, lysis and plating procedures were identical to those used in the analysis of bacterial survival. All assays were conducted in triplicate and repeated at least three times on different days.

### Quantification of *C. sakazakii* virulence gene transcription using RT-qPCR

The *C. sakazakii* strain ATCC 29544 was grown in TSB without or with TQ (0, 400, and 600 μmol/L) at 37°C to its mid-log phase. The bacteria were then centrifuged (5,000 × g, 5 min, 4°C) and re-suspended in PBS. The total RNA was extracted with the RNAprep Pure Bacteria Kit (Tiangen, Beijing, China) according to the manufacturer's protocol. To remove all DNA, the purified RNA was treated for 15 min with 30 Units of DNase I. RNA concentrations were measured with a nucleic acid and protein spectrophotometer (Nano-200; Aosheng Instrument Co., Ltd., Hangzhou, China). First-strand cDNA was synthesized from 0.5 μg of each RNA sample in a 10 μl reaction mixture using the PrimeScript™RT reagent kit (TakaRa, Kyoto, Japan) according to manufacturer directions. The primer sequences used for RT-PCR are listed in Table 1. RT-PCR was performed in a 25-μl system using SYBR® Premix Ex Taq™ II (TakaRa). The cycling conditions included 1 cycle of 95°C for 30 s, 40 cycles of 95°C for 5 s and 60°C for 30 s, and dissociation steps of 95°C for 15 s and 60°C for 30 s. All samples were analyzed in triplicate and normalized to the endogenous control (ESA_04030) gene. Samples were run on the IQ5 system (Bio-Rad Laboratories, Hercules, CA, U.S.A.) and the transcription of target genes vs. ESA_04030 gene were determined as previously described (Li et al., 2014).

**Table 1:** Minimum inhibitory concentrations of TQ against different strains of *C. sakazakii*.

**Strain**	**Origin**	**MIC (μmol/L)**
ATCC 29544	Child's throat	2,400
ATCC BAA-894	Human clinical specimen	1,800
ATCC 12868	Infant formula	3,600
ATCC 29004	Infant formula	2,400
12-2	Infant rice cereal	3,600
14-15	Infant formula	2,400
18-7	Infant rice cereal	2,400
18-8	infant formula	2,400
18-13	Infant formula	2,400
7-17	Infant formula	2,400

### Statistical analysis

Statistical analyses were performed using SPSS software (version 19.0; SPSS, Inc., Chicago, IL). The data were presented as the mean values ± *SD* and differences between means were tested by Student's *t*-test. Differences are considered significant at *P* ≤ 0.05. All experiments were performed at lease in triplicate.

## Results

### MICs and SICs

**Figure 1:**
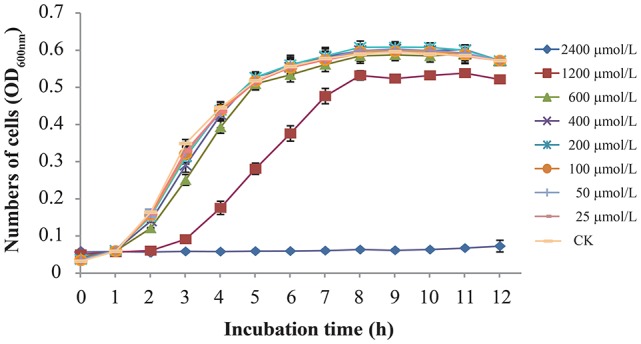
Growth of *C. sakazakii* ATCC 29544 in TSB with various concentrations of TQ. Each value represents the average of three independent measurements.

The MICs of TQ for 9 *C. sakazakii* strains are presented in Table 1. It is shown by agar dilution assay that TQ exhibited antimicrobial activity against all the tested strains, and the MICs ranged from 1,800 to 3,600 μmol/L. Broth dilution assay showed that concentrations below 600 μmol/L exhibited no growth inhibition against *C. sakazakii* ATCC 29544 (Figure 1) and were further chosen to study the effects of TQ on *C. sakazakii* virulence. Since TQ was equally effective against our three *C. sakazakii* isolates (ATCC 29544, ATCC 29004, and 7-17), only strain ATCC 29544 was selected for further studies.

### Motility

**Figure 2:**
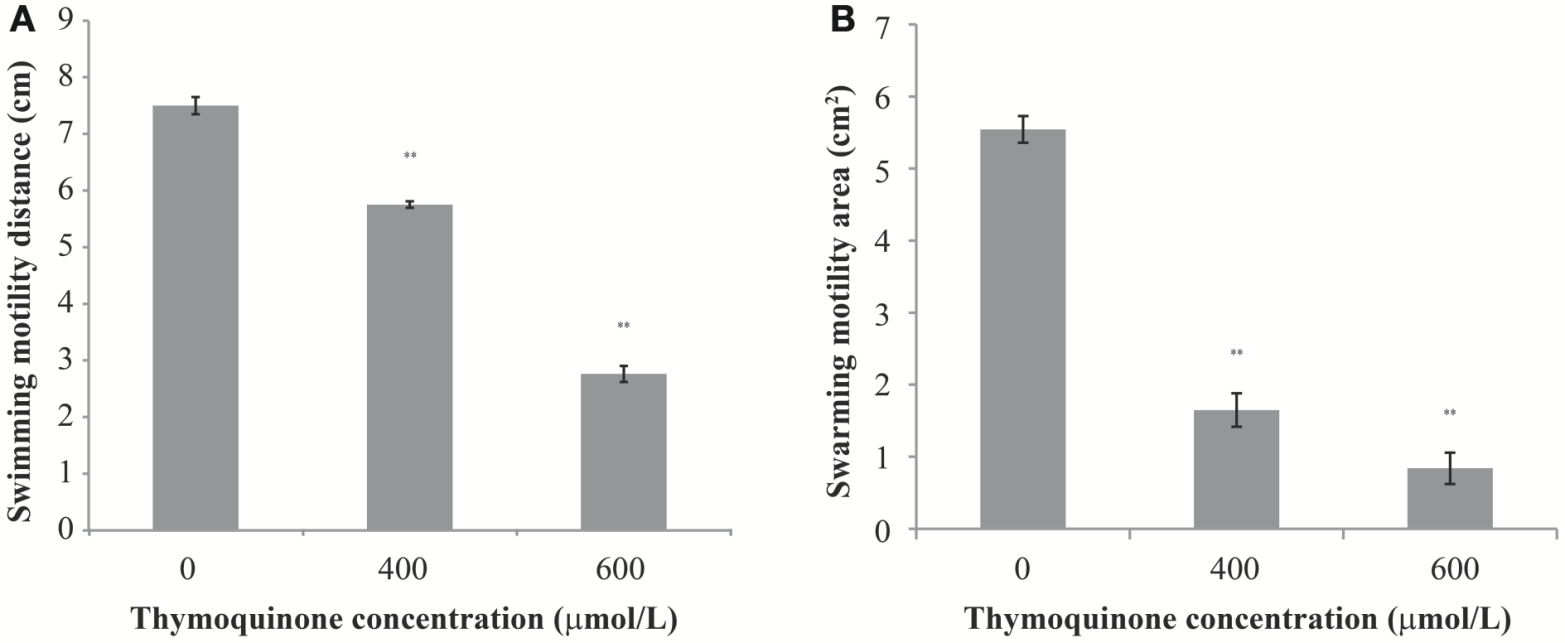
The effect of TQ on swimming motility **(A)** and swarming motility **(B)** of *C. sakazakii* ATCC 29544. Bars represent the standard deviation (*n* = 3). ^**^P ≤ 0.01 compared to control.

The effect of TQ on *C. sakazakii* motility is shown in Figure 2. TQ reduced both swimming and swarming ability of *C. sakazakii* ATCC 29544 (Figures 2A,B). The original swimming distance of *C. sakazakii* ATCC 29544 was 7.50 ± 0.15 cm. Addition of TQ at 600 and 400 μmol/L caused swimming distance reductions to 2.76 ± 0.14 cm (*P* ≤ 0.01) and 5.75 ± 0.06 cm (*P* ≤ 0.01) respectively. Swarming motility was also greatly impacted by TQ. The original swarming area of *C. sakazakii* ATCC 29544 was 5.54 ± 0.19 cm^2^. TQ at 600 and 400 μmol/L caused swarming area reductions to 0.84 ± 0.22 cm^2^ (*P* ≤ 0.01) and 1.65 ± 0.23 cm^2^ (*P* ≤ 0.01) respectively.

### TQ reduces endotoxin production in *C. sakazakii*

**Figure 3:**
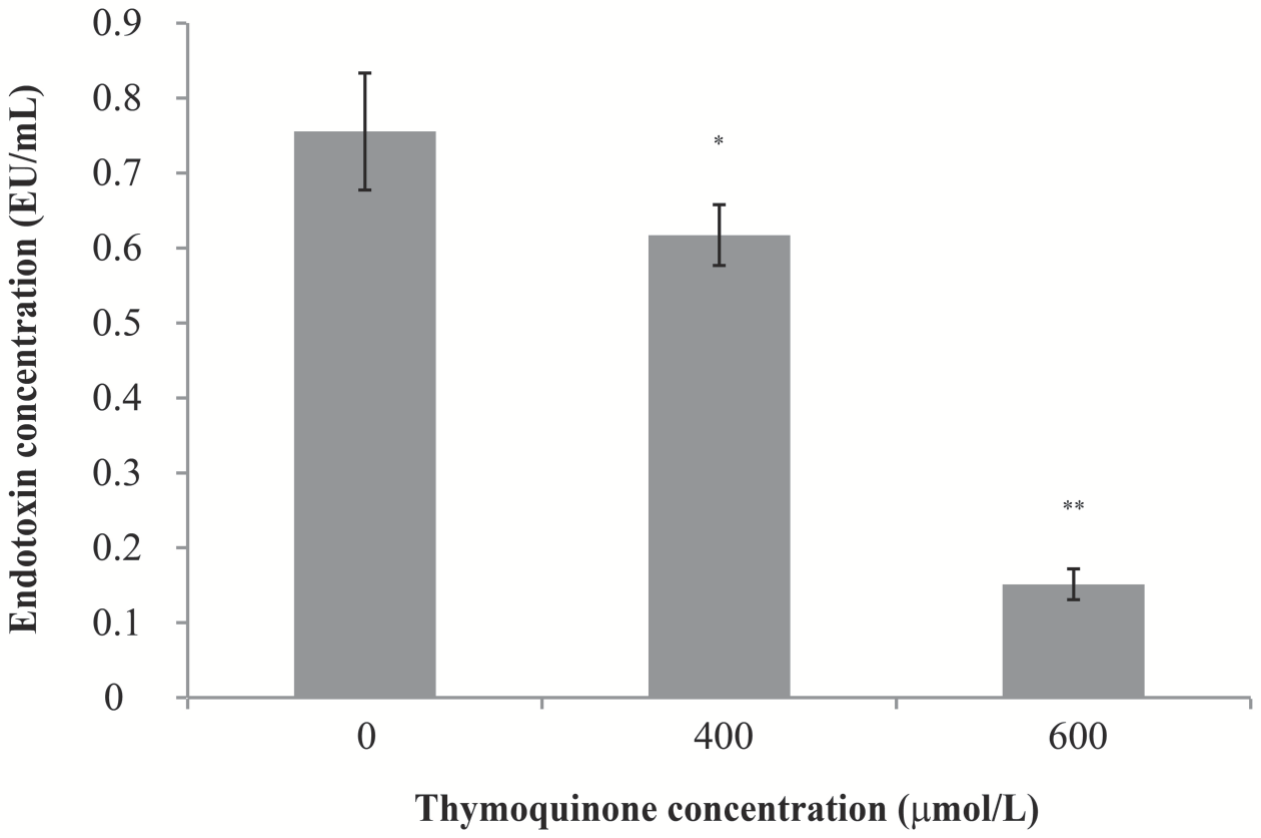
Effect of TQ on endotoxin production by *C. sakazakii* ATCC 29544. Bars represent the standard deviation (*n* = 3). ^**^P ≤ 0.01, ^*^P ≤ 0.05 compared to control.

The original endotoxin concentration of *C. sakazakii* ATCC 29544 was 0.76 ± 0.08 EU/ml (Figure 3). After the addition of TQ to the *C. sakazakii*, we observed a clear change in endotoxin concentration. Addition of TQ at 400 μmol/L caused a significant fall (*P* ≤ 0.05) in *C. sakazakii* endotoxin concentration to 0.62 ± 0.04 EU/ml. Addition of TQ at 600 μmol/L caused a further decrease in endotoxin concentration (*P* ≤ 0.01) to 0.15 ± 0.02 EU/ml.

### Anti-QS activity of TQ

**Figure 4:**
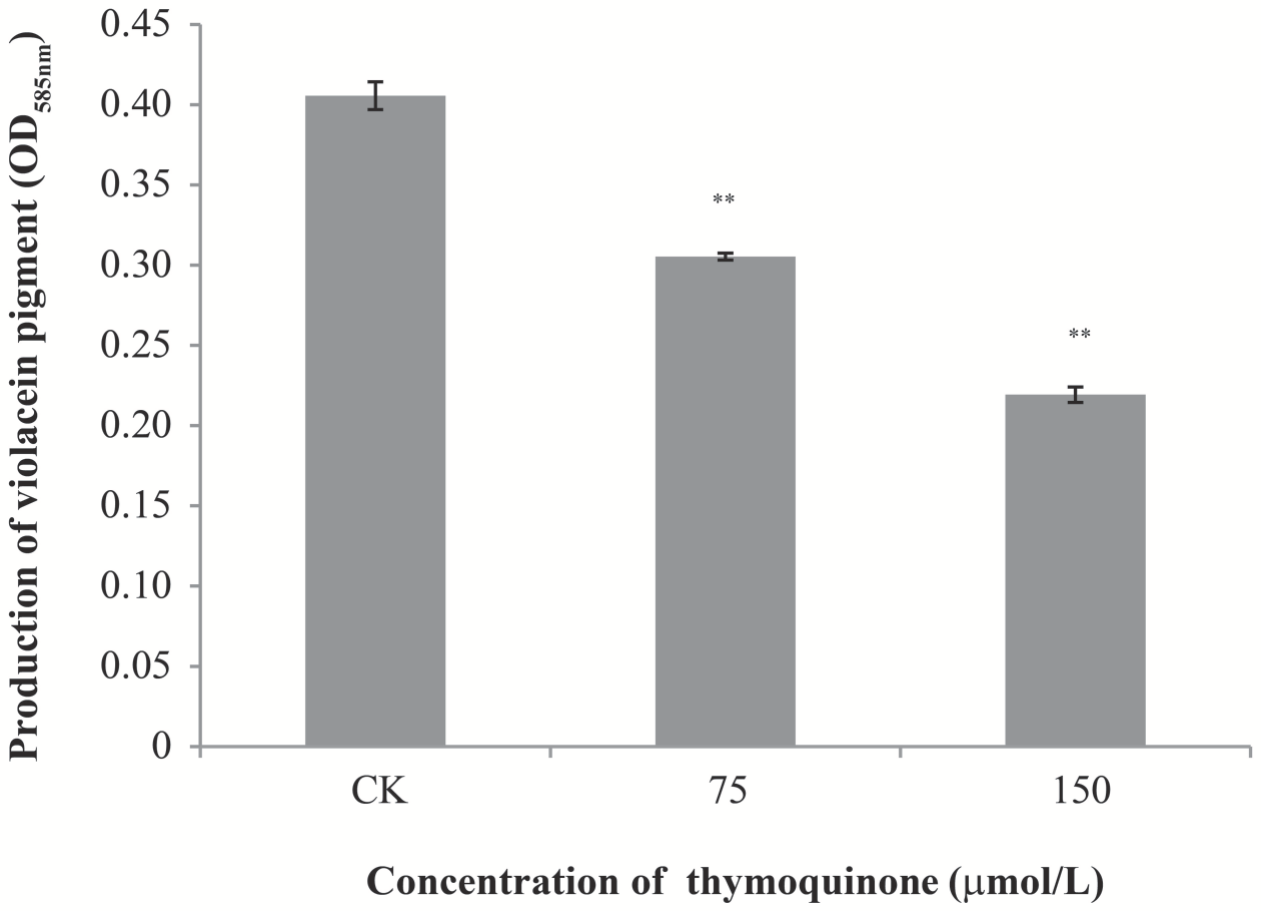
Inhibition of violacein production by *C. violaceum* ATCC 12472 at different concentrations of TQ. Bars represent the standard deviation (*n* = 3). ^**^P ≤ 0.01 compared to control.

TQ showed no growth inhibition activity against *C. violaceum* at concentrations used in this study (75 and 150 μmol/L) with a broth micro-dilution method (data not shown). As seen in Figure 4, anti-QS activity was shown when TQ was used at 75 and 150 μmol/L, as evidenced by a significant decreased production of violacein (about 75.27 and 54.07% of the control, respectively).

### Anti-biofilm formation of TQ

The anti-biofilm efficacy of TQ was investigated on *C. sakazakii* strain 7-17 grown at 12 and 25°C for 24, 48, and 72 h on microtiter plates (Table 2). Compared with the control, cells treated with TQ showed significantly less biofilm formation. For *C. sakazakii* 7-17, the biofilm formation of cells treated with 600 μmol/L TQ was inhibited by 45.6, 44.4, and 68.3% after 24, 48, and 72 h at 25°C, respectively. And TQ at 600 μmol/L reduced the initial biofilm formation by 31.5, 73.5, and 81.4% of the control level when *C. sakazakii* 7-17 grown at 12°C for 24, 48, and 72 h.

**Table 2:** Inhibition of *C. sakazakii* 7-17 biofilm formation by TQ at different concentrations at 25°C and 12°C.

**Growth conditions**	**SBF**		
	**CK**	**TQ at 400 μmol/L**	**TQ at 600 μmol/L**
**25°C TIME (h)**			
24	2.48 ± 0.13	1.42 ± 0.22^b^	1.35 ± 0.18^b^
48	2.25 ± 0.15	1.54 ± 0.10^b^	1.25 ± 0.11^b^
72	2.52 ± 0.20	1.63 ± 0.18^b^	0.80 ± 0.14^b^
**12°C TIME (h)**			
24	1.08 ± 0.26	0.82 ± 0.09	0.74 ± 0.06
48	2.79 ± 0.21	1.43 ± 0.12^b^	0.74 ± 0.06^b^
72	2.10 ± 0.09	0.34 ± 0.08^b^	0.39 ± 0.07^b^

b *P ≤ 0.01 compared to control*.

### Adhesion to and invasion of HT-29 cells

**Figure 5:**
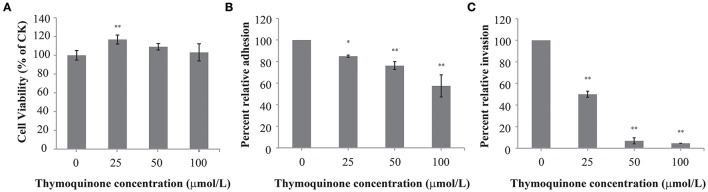
Effect of TQ on HT-29 cells viability **(A)**; Effect of TQ on adhesion **(B)** and invasion **(C)** of HT-29 cells by *C. sakazakii* ATCC 29544. Bars represent the standard deviation (*n* = 3). ^*^P ≤ 0.05, ^**^P ≤ 0.01 compared to control.

TQ at concentrations of up to 100 μmol/L did not affect cell viability (Figure 5A). Therefore, TQ at concentrations of 25, 50, and 100 μmol/L were used in further studies. TQ at 25, 50, and 100 μmol/L inhibited adhesion of *C. sakazakii* to 85.0, 76.3, and 57.5%, respectively compared to the control (Figure 5B). TQ was also effective in inhibiting (*P* ≤ 0.01) the ability of *C. sakazakii* ATCC 29544 to invade HT-29 cells (Figure 5C). The invasiveness of *C. sakazakii* ATCC 29544 treated with 25, 50, and 100 μmol/L TQ was reduced to 50.0, 6.8, and 4.6% of the control, respectively.

### Intracellular survival in RAW 264.7

**Figure 6:**
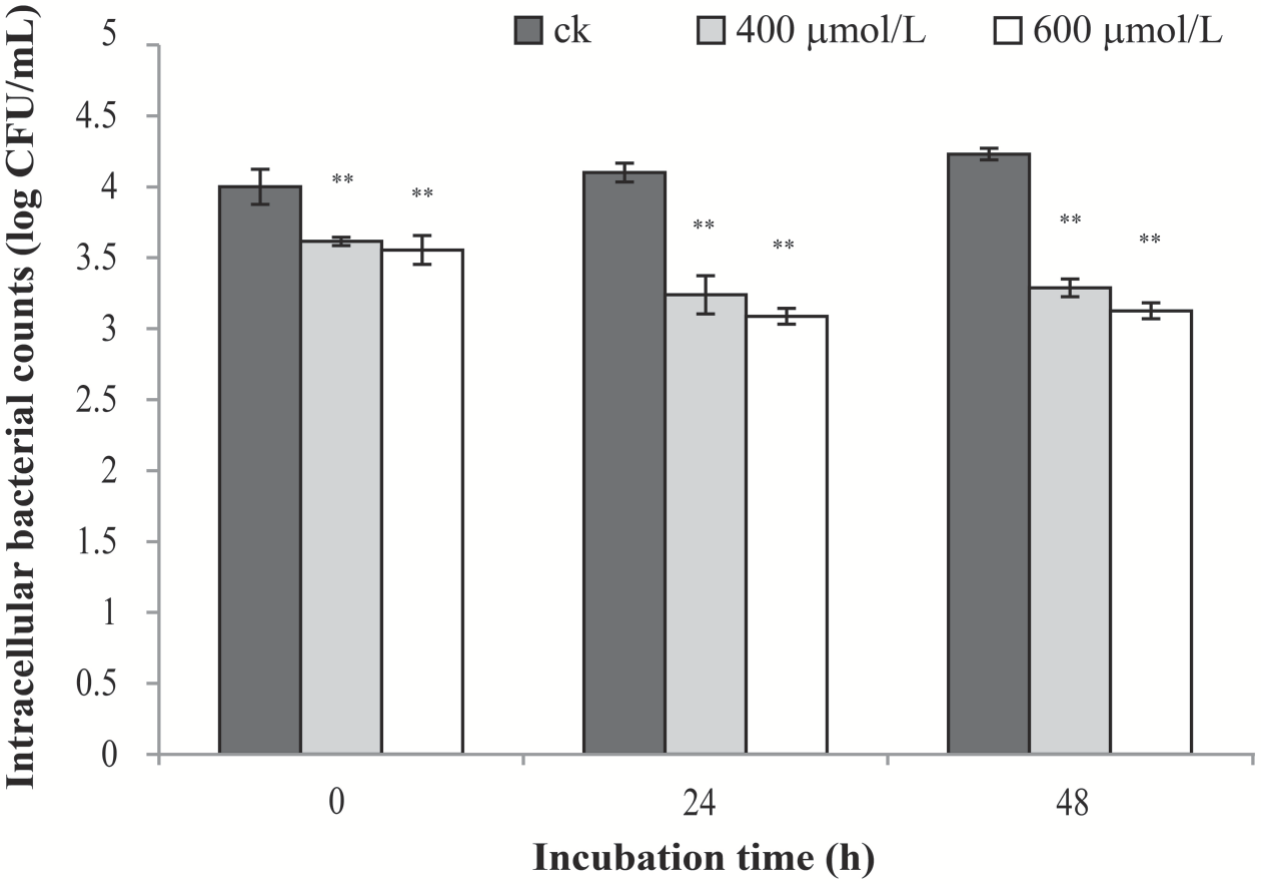
Effect of TQ on bacterial intracellular survival and replication of *C. sakazakii* ATCC 29544 within macrophages. Bars represent the standard deviation (*n* = 3). ^**^P ≤ 0.01 compared to control.

TQ-untreated *C. sakazakii* ATCC 29544 was able to survive well in macrophage (Figure 6). The results showed that TQ at concentrations of 400 and 600 μmol/L were able to significantly (*P* ≤ 0.01) decrease intracellular survival of *C. sakazakii* in the macrophages during 48 h when compared to the control.

### Virulence-associated genes

RT-qPCR demonstrated that TQ significantly decreased the transcription of sixteen virulence-related genes in *C. sakazakii* (Table 3). TQ down-regulated the transcription of *fliD, flhD*, and *flgJ* genes (critical for flagella regulation) to various degrees. Other down-regulated genes were *ompA* (outer membrane protein A), *ompX* (outer membrane protein X), *uvrY* (adherence and invasion), *motA, motB* (flagellar motor protein), *sod* (survival in macrophages), *bcsA* (cellulose synthase catalytic subunit), *bcsG* (cell biosynthesis and biofilm formation), *lpx, wzx* (LPS biosynthesis), *luxR* (LuxR family transcriptional regulator), *galE* (colanic acid synthesis), and *kpsT* (K-antigen synthesis) (Table 3).

**Table 3:** Differentially expressed virulence-related genes in *C. sakazakii* ATCC 29544 grown in TSB with TQ at different concentrations.

**Target gene**	**Sequence of primers(5′-3′)^c^**	**Relative gene expression**	
		**400 μmol/L**	**600 μmol/L**
ESA_04030	F, CCAGGGCTACACACGTGCTA	1	1
	R, TCTCGCGAGGTCGCTTCT		
*bcsA*	F, CACGATGGTGGCGTTGTTC	−1.74 ± 0.3^a^	−4.62 ± 1.49^a^
	R, CCTTTGGCGGTGACGTTAA		
*bcsG*	F, ACGACTGGCTGAACAGCTTTTAC	−1.32 ± 0.37	−4.74 ± 0.23^b^
	R, GCCGGGAAGGTTGTCTGA		
*flhD*	F, CGATGTTTCGCCTGGGAAT	−1.10 ± 0.06^a^	−2.66 ± 0.16^b^
	R, AGAGTCAGGTCGCCCAGTGT		
*fliD*	F, AAAACCGCAACATGGAATTCA	−1.78 ± 0.13^b^	−7.29 ± 0.57^b^
	R, CCGCAAACGCGGTATTG		
*flgJ*	F, GACGGCGGGCAAAGG	−1.12 ± 0.62	−3.17 ± 0.52^b^
	R, GCCGCCCATCTGTTTGAC		
*motA*	F, GGTGTGGGTGCGTTTATCGT	−1.06 ± 0.01^a^	−2.83 ± 0.38^b^
	R, GCCTTCAGCGTGCCTTTG		
*motB*	F, ACGGCTCGTGGAAAATCG	−1.09 ± 0.02^a^	−2.54 ± 0.21^b^
	R, CCAGGAAGAAGGCCATCATG		
*luxR*	F, TGTGCGTTCGCCATCCT	−1.23 ± 0.10^a^	−4.91 ± 1.81^a^
	R, TGGTGTGCAGCGTCAGTTTT		
*lpxB*	F,GCACGACACTTTCCGTAAACTG	−1.05 ± 0.02^b^	−1.69 ± 0.12^b^
	R,CGCCTGTTCATCGGCATT		
*ompA*	F,GGCCGCATGCCGTATAAA	−2.15 ± 0.01^b^	−2.70 ± 0.37^b^
	R,GCTGTACGCCCTGAGCTTTG		
*ompX*	F,GTCTTTCAGCACTGGCTTGTGT	−1.51 ± 0.16^b^	−4.13 ± 0.68^b^
	R,GGTGCCAGCAACAGCAGAA		
*sod*	F,CGAATCTGCCGGTTGAAGA	−1.36 ± 0.14^a^	−1.99 ± 0.08^b^
	R,CTTGTCCGCCGGAACCT		
*uvrY*	F,GCGAGGACGCCATCAAAT	−1.57 ± 0.25^a^	−2.12 ± 0.48^a^
	R,ATCCATCAGCACCACATCCA		
*wzx*	F,TGCTTGGGCAGGTACAAAGTG	−1.39 ± 0.08^b^	−4.28 ± 0.90^b^
	R,CCCTACGGGTGCAGTCACA		
*galE*	F,CTTGAGTATTACGACAACAACG	−3.39 ± 0.29^b^	−3.94 ± 0.35^b^
	R,GAAACTTTCGACATAAGGGAT		
*kpsT*	F,ATTGGCGGGACGGATAA	−2.50 ± 0.19^b^	−4.85 ± 0.33^b^
	R,TCGTCCACCAGGTAGTAGTCA		

a P ≤ 0.05,

b P ≤ 0.01,

c *F, forward; R, reverse*.

## Discussion

The motility is considered as a virulence factor for intestinal and urogenital-tract pathogens (Manson, 1992). To establish infection and incite disease development, bacteria first have to attach and adhere to a surface, either abiotic or biotic. The biofilm of *C. sakazakii* on abiotic surfaces in food and medical sectors constitutes a great public health concerns (Kim et al., 2006). Flagella seem to play a role in the initial phases of biofilm development in *C. sakazakii*, in attachment as well as further formation. In addition, motility also plays a role in adhesion to both plant and animal tissues. Hartmann et al. (2010) observed that the absence of flagella greatly reduced the adhesion capacity, suggesting that flagella are important for *Cronobacter* adhesion to biotic surfaces. In this study, we observed that the sub-inhibitory concentration of thymoquinone obviously reduced the swimming and swamming motility area of *C. sakazakii*. To gain further insights into the effect of TQ on *C. sakazakii* motility, three key flagellar structure or biosynthesis genes (Hartmann et al., 2010) were selected to test their transcription. *flhD* encodes the transcriptional activator FlhD. *fliD* encodes the flagellar capping protein, *flgJ* encodes a muramidase gene involved in the flagellar rod assembly protein FlgJ. Interestingly, TQ suppressed *flhD, fliD*, and *flgJ* at transcriptional levels (Table 3), the loss of functionality of the flagellum could be the possible mechanism by which TQ suppresses swimming and swarming motility. These findings suggest that TQ can reduce the capacity of *C. sakazakii* to access to a surface actively and further establish effective infection.

It has been reported that some *C. sakazakii* strains are able to form biofilms on glass, silicone, stainless steel, polyvinyl chloride, and enteral feeding tubes in various media (Kim et al., 2006). Biofilms protect the embedded cells against detachment by flow shear and help them resist adverse conditions, such as presence of antimicrobials and biocides (Jung et al., 2013). Because of persistence and high resistance properties of mature biofilm there is an increasing interest to search for substances, which inhibit specific processes in the initial phase of biofilm formation. Szczepanski and Lipski (2014) reported that thyme, oregano, and cinnamon essential oil are able to inhibit the biofilm formation of the genera *Stenotrophomonas, Acinetobacter*, and *Sphingomonas* which were isolated from authentic biofilms in the food industry. Our results showed that TQ inhibited biofilm formation of strong biofilm producer on microtiter plates at 12° and 25°C in 72 h. Cellulose has been identified and characterized as an extracellular matrix component present in the biofilm of *C. sakazakii* (Grimm et al., 2008). The presence of TQ decreased the transcription level of *bcsA*, which encodes the catalytic subunit of the cellulose synthase and *bcsG*, which encodes the conserved hypothetical protein (Table 3). Therefore, it is hypothesized that TQ may inhibit *C. sakazakii* biofilm formation by inhibition of cellulose and flagella production.

Bacterial quorum sensing has been reported to control virulence gene expression with the presence of small signal molecules in numerous micro-organisms (Defoirdt et al., 2013). AHL, as the autoinducer signal in QS, accumulates in the medium and activates the transcriptional activator *luxR* to control a variety of physiological processes (Winzer and Williams, 2001). AHL-controlled functions in bacteria include conjugation, secretion of virulence factors, motility, pigment production, biofilm formation, and bioluminescence (Henke and Bassler, 2004). In this study, we used biomarker strain *C. violaceum* ATCC 12472, which can respond exogenous short chain AHL and produce violacein (Lichstein and Sand, 1945). TQ inhibited the production of AHL-regulated violacein pigment in *C. violaceum* possibly through disruption of QS signaling systems. Similarly, Vasavi et al. (2014) reported that inhibition of violacein production by the *Psidium guajava* L. flavonoids in *C. violaceum* indicated possible anti-QS activity. Musthafa et al. (2010) showed *Ananas comosus, Musa paradiciaca*, and *Ocimum sanctum* extracts significant reducted violacein production in *C. violaceum* and can be potentially developed as an alternative to antibiotic compounds to prevent AHL-mediated bacterial infection. In addition, RT-PCR assays also confirmed that TQ down-regulated the transcription of the *luxR* gene in *C. sakazakii* in this study.

The binding of pathogens to intestinal epithelial cells is one of the prerequisites for systemic infection. Hunter et al. (2008) found that *C. sakazakii* is able to bind to enterocytes in rat pups at the tips of villi, induces disruption of tight junctions between enterocytes and ultimately causes apoptosis of the cells. Kim and Loessner (2008) demonstrated that *C. sakazakii* exhibited significantly higher invasiveness than other *Enterobacter* species. The adhesion and invasion of *C. sakazakii* play an important role for itself to translocate through the intestinal lumen into the blood circulation and establish a systemic infection with symptoms such as bacteremia and necrotizing enterocolitis. Outer membrane proteins OmpX and OmpA were shown to play critical roles in *C. sakazakii* invasion through both apical side and basolateral side of the host cells, and they are responsible for *C. sakazakii* translocation into the deeper organs (i.e., liver and spleen) (Kim et al., 2010). The *uvrY* gene regulates the ability of *E. coli* to adhere, invade, persist within tissues (Herren et al., 2006). Our results demonstrate that *C. sakazakii* adhesion and invasion of HT29 cells were inhibited by TQ, and TQ significantly downregulated *ompA, ompX*, and *uvrY* expression. Likewise, Amalaradjou et al. (2014) reported that trans-cinnamaldehyde was effective in markedly reducing the attachment and invasion of rat intestinal epithelial cells by *C. sakazakii*.

The ability of pathogens to enter, persist and/or grow within macrophages is also important for infection. Almajed and Forsythe (2016) reported that some ST4 and ST1 *C. sakazakii* strains were able to survive for up to 72 h of incubation and multiply significantly within human macrophages cell line U937. In this study, *C. sakazakii* strain ATCC 29544 (ST1) showed the ability to persist within macrophages cell line RAW 264.7 for up to 48 h of incubation. Additionally, our results showed TQ reduced the persistence of *C. sakazakii* with RAW 264.7 cells. Macrophages and polymorphonuclear cells typically engulf pathogens and also effectively kill nearby organisms by the release of reactive oxygen species. Superoxide dismutases in bacterium provides the capacity to neutralize toxic levels of ROS produced by the host (Lynch and Kuramitsu, 2000). In this study, we found that TQ significantly downregulated transcription of *sod*, which codes for the superoxide dismutases of *C. sakazakii*. Our results indicated that TQ decreased the chances of *C. sakazakii* to use macrophages as a vehicle for escaping from the immune response and invading the other body organs.

Endotoxin (also known as lipopolysaccharide, LPS), a major amphophilic molecule embedded in the outer envelope of Gram-negative bacteria, is a potent immune activator and essential virulence factor of Gram-negative bacteria. LPS causes release of numerous host proinflammatory cytokines and activates the complement cascade and the coagulation cascade which play an important role in the virulence of Gram-negative pathogens (Das, 2000). Sivamaruthi et al. (2015) reported that *C. sakazakii* LPS is sufficient to affect the *Caenorhabditis elegans* pharyngeal pumping rate, brood size and cause lethality. Townsend et al. (2008) demonstrated the influence of endotoxin on increased translocation of intestinal bacteria in the neonatal rat. Here we proved that TQ inhibited the production of endotoxin in *C. sakazakii* and downregulated the expression of *lpx* and *wzx*, which are essential for the synthesis of LPS in Gram-negative bacteria.

In this study, we demonstrated that SICs of TQ decreased crucial virulence related traits in *C. sakazakii* ATCC 29544 including motility, expression of certain virulence genes, adhesion and invasion to HT-29 cells and intracellular survival of bacterial cells within the RAW 264.7 macrophage cells. In addition, TQ significantly inhibited biofilm formation of *C. sakazakii* 7-17 and interfered with AHL-dependent QS system in *C. violaceum*. The effects of TQ on other *C. sakazakii* strains and *in vivo C. sakazakii* infection model need further investigation before TQ could be potentially developed as an alternative strategy to control the diseases attributed to *C. sakazakii*.

## Author contributions

CS, CY, and XX conceived and designed the experiments. YSui, YSun, and DG performed the experiments. YC and TJ analyzed the data. XP and LM contributed reagents, materials, analysis tools. CS and CY wrote the manuscript.

### Conflict of interest statement

The authors declare that the research was conducted in the absence of any commercial or financial relationships that could be construed as a potential conflict of interest.
