# Corrigendum: Thymoquinone Inhibits Virulence Related Traits of *Cronobacter sakazakii* ATCC 29544 and Has Anti-biofilm Formation Potential

**DOI:** 10.3389/fmicb.2018.00290

**Published:** 2018-02-20

**Authors:** Chao Shi, Chunhong Yan, Yue Sui, Yi Sun, Du Guo, Yifei Chen, Tong Jin, Xiaoli Peng, Linlin Ma, Xiaodong Xia

**Affiliations:** ^1^College of Food Science and Engineering, Northwest A&F University, Yangling, China; ^2^College of Animal Science and Technology, Northwest A&F University, Yangling, China; ^3^School of Life Science and Technology, Xi'an Jiaotong University, Xi'an, China; ^4^Xi'An Yurun Agricultural Products Global Sourcing Co., LTD., Xi'an, China

**Keywords:** *Cronobacter sakazakii*, Thymoquinone, sub-inhibitory concentration, HT-29 cells, Virulence Factors

In the first paragraph in the “Introduction” of this article, the number of *Cronobacter* species was incorrectly described as ten. Actually, *Cronobacter pulveris, Cronobacter helveticus* and *Cronobacter turicensis* were removed from the genus in 2014.

The original sentence should be corrected as follows: *Cronobacter* spp. is currently considered to consist of 7 species: *Cronobacter sakazakii, C. malonaticus, C. universalis, C. dublinensis, C. muytjensii, C. condiment*, and *C. zurichensis* (Stephan et al., 2014), among which *C. sakazaii* is one of the two group 1 clinically relevant species that form the majority of the clinical isolates. The authors regret this error.

The original article has been updated.

## Conflict of interest statement

The authors declare that the research was conducted in the absence of any commercial or financial relationships that could be construed as a potential conflict of interest.
